# The relative ages of ectomycorrhizal mushrooms and their plant hosts estimated using Bayesian relaxed molecular clock analyses

**DOI:** 10.1186/1741-7007-7-13

**Published:** 2009-03-10

**Authors:** David S Hibbett, P Brandon Matheny

**Affiliations:** 1 Biology Department, Clark University, Worcester, Massachusetts 01610, USA; 2 Department of Ecology and Evolutionary Biology, University of Tennessee, Knoxville, Tennessee 37996, USA

## Abstract

**Background:**

Ectomycorrhizae (ECM) are symbioses formed by polyphyletic assemblages of fungi (mostly Agaricomycetes) and plants (mostly Pinaceae and angiosperms in the rosid clade). Efforts to reconstruct the evolution of the ECM habit in Agaricomycetes have yielded vastly different results, ranging from scenarios with many relatively recent origins of the symbiosis and no reversals to the free-living condition; a single ancient origin of ECM and many subsequent transitions to the free-living condition; or multiple gains and losses of the association. To test the plausibility of these scenarios, we performed Bayesian relaxed molecular clock analyses including fungi, plants, and other eukaryotes, based on the principle that a symbiosis cannot evolve prior to the origin of both partners. As we were primarily interested in the relative ages of the plants and fungi, we did not attempt to calibrate the molecular clock using the very limited fossil record of Agaricomycetes.

**Results:**

Topologically constrained and unconstrained analyses suggest that the root node of the Agaricomycetes is much older than either the rosids or Pinaceae. The Agaricomycetidae, a large clade containing the Agaricales and Boletales (collectively representing 70% of Agaricomycetes), is also significantly older than the rosids. The relative age of Agaricomycetidae and Pinaceae, however, is sensitive to tree topology, and the inclusion or exclusion of the gnetophyte *Welwitschia mirabilis*.

**Conclusion:**

The ancestor of the Agaricomycetes could not have been an ECM species because it existed long before any of its potential hosts. Within more derived clades of Agaricomycetes, there have been at least eight independent origins of ECM associations involving angiosperms, and at least six to eight origins of associations with gymnosperms. The first ECM symbioses may have involved Pinaceae, which are older than rosids, but several major clades of Agaricomycetes, such as the Boletales and Russulales, are young enough to have been plesiomorphically associated with either rosids or Pinaceae, suggesting that some contemporary ECM partnerships could be of very ancient origin.

## Background

Ectomycorrhizae (ECM) are symbiotic associations that involve fungi and many of the dominant trees of both temperate and tropical forests. Reconstructing the origins of ECM associations is important to understand the evolution of terrestrial ecosystems, but this task is made difficult by extensive homoplasy in the evolution of these symbioses in both fungi and plants. This study focuses on the evolution of the ECM habit in the Agaricomycotina (Basidiomycota), which contains the vast majority of ECM-forming fungi. Most studies on this subject have used phylogenetic approaches coupled with ancestral state reconstruction (ASR) [1-5]. Here, we use molecular clock analyses to estimate the relative ages of major clades of Agaricomycotina and their potential plant hosts. We focus on key nodes in the fungal phylogeny that subtend clades containing ECM and saprotrophic taxa, and ask whether it is plausible that the ancestors at these nodes could have been partners in ancient ECM associations.

**Figure 1:**
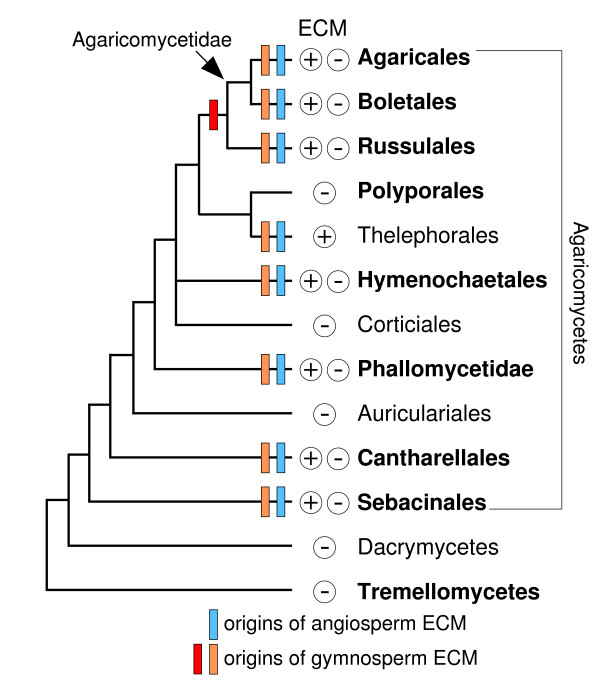
**Simplified phylogeny of Agaricomycotina**. Clades sampled are indicated in bold font. Presence/absence of ectomycorrhizae (ECM) taxa is indicated by + and - symbols (Gloeophyllales, Trechisporales, and Atheliales are not shown). Blue bars: minimum origins of ECM symbioses with angiosperms. Orange bars: minimum origins of ECM symbioses with gymnosperms under unconstrained topology and constrained topology excluding *Welwitschia*. Red bar: alternative minimum origin of ECM with gymnosperms in Agaricomycetidae + Russulales, under constrained topology retaining *Welwitschia*.

The Agaricomycotina comprises three major clades, the Agaricomycetes, Tremellomycetes, and Dacrymycetes (Figure 1). The Agaricomycetes is by far the largest, with 20,951 described species (98% of Agaricomycotina) [6], including diverse ECM-forming species and saprotrophs, as well as lesser numbers of plant pathogens, mycoparasites, insect symbionts, and lichens. The Tremellomycetes and Dacrymycetes (yeasts and jelly fungi) contain mycoparasites, animal pathogens, and wood-decayers and they form a paraphyletic grade at the base of the Agaricomycotina, suggesting that the plesiomorphic condition of the Agaricomycotina is non-ECM [7-10].

The Agaricomycetes can be further divided into 14 independent clades, eight of which contain ECM species (Agaricales, Boletales, Russulales, Thelephorales, Hymenochaetales, Phallomycetidae, Cantharellales (including 'Tulasnellales'), and Sebacinales). Except for the Thelephorales, all of the ECM-containing clades of Agaricomycetes also contain saprotrophic species (Figure 1). The pattern of transformations between ECM and saprotrophic lifestyles in the Agaricomycetes is controversial. Hibbett et al [3] reconstructed the evolution of the ECM habit using phylogenetic trees containing 161 species from across the Agaricomycetes. ASR using equally weighted parsimony and maximum likelihood (ML) methods suggested that the ancestor of the Agaricomycetes was saprotrophic, and that there have been 7 to 13 independent gains of the ECM habit and three to nine losses (reversals to the saprotrophic condition) across the Agaricomycetes. The inferred reversals to the free-living condition were surprising, in part because they suggested that mutualisms are not stable endpoints in evolution. The largest concentration of 'secondarily derived' saprotrophs inferred by Hibbett et al [3] was in the Agaricomycetidae, a large clade containing the Agaricales (13,233 described species) and Boletales (1316 species), which collectively represent about 70% of Agaricomycetes. The analysis of Hibbett et al [3] suggested that the most recent common ancestor of the Agaricomycetidae was an ECM-forming species, implying that all of the saprotrophic species of Agaricomycetidae were ultimately derived from symbiotic taxa.

Several recent analyses have cast doubt on the occurrence of reversals from ECM to saprotrophic lifestyles. Bruns and Shefferson [2] repeated the parsimony based ASR of Hibbett et al [3], using the same trees, but with a two-to-one weighting of losses vs. gains. Under this weighting regime, all but one reversal disappeared, and that reversal (involving *Lentaria byssoides *in the Phallomycetidae) could be an artifact due to limited taxon sampling or erroneous character scoring. Analyses focused within the Agaricales and Boletales have also suggested that there have been multiple origins of the ECM habit and no losses [1,5]. Yet another scenario for the evolution of ECM in Agaricomycotina was proposed by Weiss and colleagues [7,11], who suggested that the ancestor of all the Agaricomycetes might have been an ECM species, with many later reversals to the free-living condition. This conclusion was based on the discovery that the Sebacinales, which appears to be the sister group of all other Agaricomycetes, includes many ECM-forming species. In short, tree-based reconstructions of ancestral states have yielded vastly different interpretations of the evolution of ECM in Agaricomycetes, ranging from scenarios with many relatively recent gains and no losses [1,2,5], a single ancient gain and many subsequent reversals [11], or multiple gains and losses [3].

As an alternative to ASR, molecular clock analyses can be used to identify branches in the phylogeny in which the evolution of ECM could have been possible, based on the principle that a symbiosis cannot arise prior to the origin of both partners. Molecular clock analyses are complicated by heterogeneity in rates of molecular evolution and the need for calibration. The oldest unambiguous Agaricomycete fossils are *Quatsinoporites cranhamii *ca. 130 to 125 Ma [12], which is probably in the Hymenochaetales or Polyporales, and *Archaeomarasmius leggeti *ca. 90 to 94 Ma [13], which is most likely in the Agaricales, possibly in the 'Marasmioid clade' [5]. The oldest fossils of ECM roots are from the Middle Eocene, ca. 50 Ma [14]. The paucity of Agaricomycete fossils, and uncertainty about their taxonomic placements, severely limit their utility for calibration purposes. The sensitivity of fungal molecular clock analyses to the taxonomic assignments of fossils was demonstrated by Taylor and Berbee [15], who used the 400 million-year-old ascomycete *Paleopyrenomycites devonicus *to calibrate a phylogeny of the fungi. *Paleopyrenomycites devonicus *has been classified in the Sordariomycetes, but Taylor and Berbee argued that it could also be a member of the Dothideomycetes, Chaetothyriomycetes, or Taphrinomycotina (all are Ascomycota). Depending on the placement of *P. devonicus*, the common ancestor of Dikarya (the clade containing Basidiomycota and Ascomycota) was estimated to be anywhere from 452 to 1489 million years old [15]. Three other molecular clock studies that analyzed datasets with 37 to 129 genes have estimated the Dikarya to be 727 to 1208 million years old [16-18]. The age of plants based on molecular clock analyses is similarly ambiguous, having been estimated to be from 425 to 703 million years old [19,20]. These discrepancies indicate that it is hazardous to compare absolute age estimates for clades taken from different studies.

The goal of our study was to place bounds on reasonable reconstructions of the evolution of the ECM habit in the Agaricomycetes by estimating the relative ages of selected fungal clades and their potential ECM hosts. We performed Bayesian relaxed molecular clock analyses [21,22] of a dataset containing genes encoding two subunits of RNA polymerase II (RPB1 and RPB2) and nuclear large and small subunit ribosomal RNA from 69 species, including fungi, plants, and other eukaryotes (Additional file 1), and we assessed which nodes in the fungal phylogeny are young enough to have been involved in ECM associations. As we were interested only in the relative ages of the plant and fungal taxa, we did not attempt to calibrate the molecular clock using the limited fungal fossil record.

## Results

**Figure 2:**
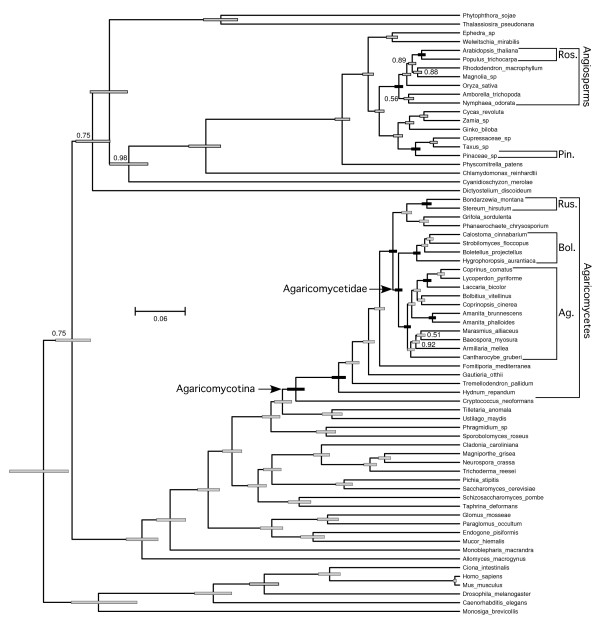
**Chronogram of eukaryotes emphasizing Agaricomycetes and potential ectomycorrhizal host plants from topologically unconstrained relaxed clock analysis**. Posterior probabilities of nodes were 1.0 except where indicated otherwise. Node bars are 95% highest posterior density intervals of node heights, expressed in average number of substitutions per site. Black node bars correspond to groups in Figure 5 and Table 1.

**Figure 3:**
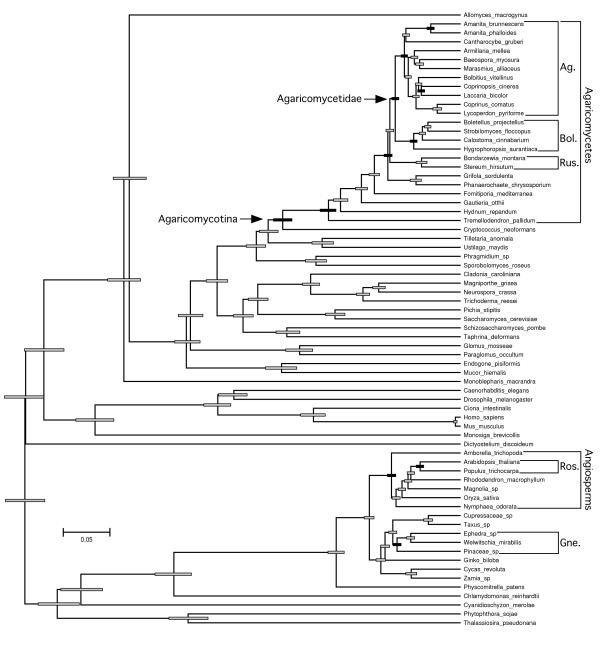
**Chronogram of eukaryotes emphasizing Agaricomycetes and potential ectomycorrhizal host plants from constrained relaxed clock analysis retaining *Welwitschia mirabilis***. Symbols as in Figure 2.

**Figure 4:**
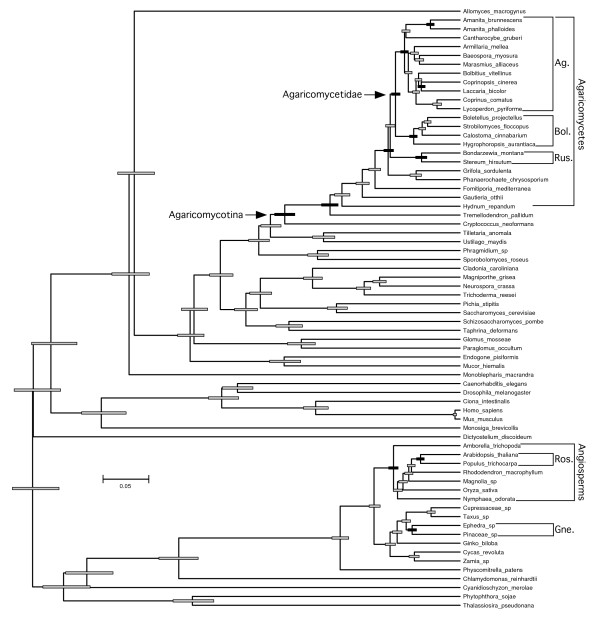
**Chronogram of eukaryotes emphasizing Agaricomycetes and potential ectomycorrhizal host plants from constrained relaxed clock analysis excluding *Welwitschia mirabilis***. Symbols as in Figures 2 and 3.

An unconstrained relaxed molecular clock analysis returned a topology that is largely consistent with prior multilocus phylogenies of fungi and other eukaryotes, with several exceptions: 1) the Polyporales is placed as the sister group of the Russulales; 2) the opisthokonts are paraphyletic, with *Dictyostelium *as the sister group of the rhodophyte-viridiplantae clade; 3)*Amborella *and *Nymphaea *form a clade that is the sister group to the remaining angiosperms; and, 4) gnetophytes are placed as the sister group of the rest of the seed plants (Figure 2). The latter feature contradicts several multilocus studies which suggest that the gnetophytes are the sister group of the Pinaceae, the so-called gnepine hypothesis [23-25]. To assess the impact of the topology on relative age estimates, we performed a pair of constrained analyses, which reflect a consensus phylogeny based on multilocus analyses of fungi [4,10,26], plants [23-25,27-29], and other eukaryotes [30,31]. The two constrained analyses differed only in the inclusion or exclusion of the gnetophyte *Welwitschia mirabilis *(Figures 3 and 4).

We evaluated the relative ages of plant and fungal clades on all three trees, considering clades with non-overlapping 95% highest posterior density ranges (HPDs) of node heights to be significantly different in age. Within the fungi, we focused on the root nodes (most recent common ancestors) of the Agaricomycetes, Agaricomycetidae, Agaricales, Boletales, and Russulales, while in the plants we focused on the root node of the rosids and the stem node of the Pinaceae.

**Figure 5:**
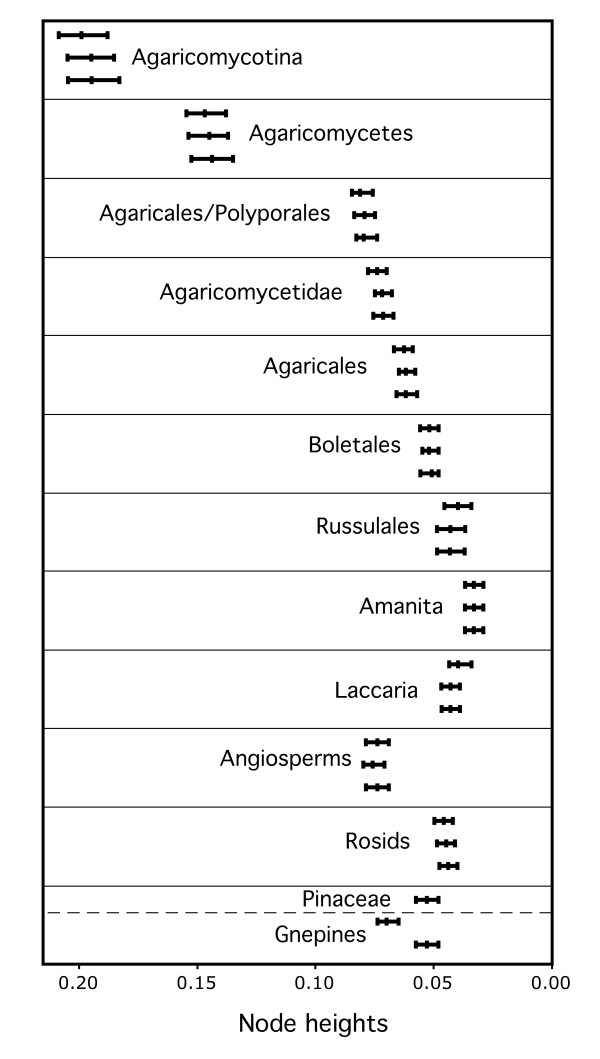
**Median and 95% highest posterior density ranges of node heights of clades of Agaricomycetes and potential host plants**. Top bar in each group is from the unconstrained analysis, middle bar is from the constrained analysis retaining *Welwitischia*, and lower bar is from the constrained analysis excluding *Welwitischia*.

**Figure 6:**
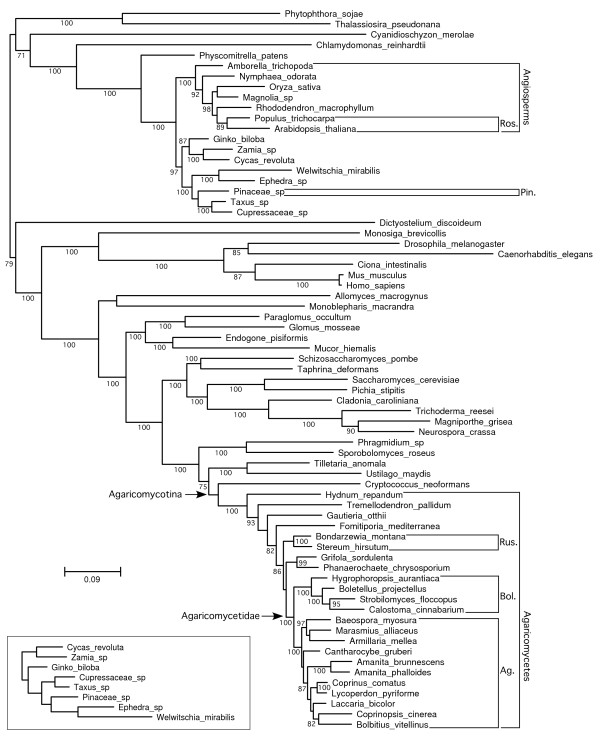
**Phylograms of eukaryotes emphasizing Agaricomycetes and potential ectomycorrhizal host plants from non-clock maximum likelihood analyses implemented in RAxML**. The large tree is the optimal topology obtained from a topologically unconstrained maximum likelihood (ML) analysis. Numbers along branches are frequencies from 100 bootstrap replicates. The small inset tree is the gymnosperm clade from a topologically constrained ML analysis (the entire dataset was analyzed, but only the gymnosperm clade is shown). Both trees are drawn to the same scale. Symbols as in Figures 2 to 4.

In all three analyses, the rosids were resolved as being significantly younger than the Agaricomycetes and Agaricales, overlapping in age with the Russulales, and marginally younger than the Boletales (that is, 95% HPDs of node heights are barely overlapping) (Figure 5; Table 1). All three analyses also suggest that the Pinaceae is significantly younger than the most recent common ancestor of the Agaricomycetidae and Polyporales, and older than, or overlapping in age with, the Boletales and Russulales (Figure 5; Table 1). In contrast, the relative age of the Pinaceae and Agaricomycetidae and Agaricales was sensitive to tree topology and taxon sampling. In the unconstrained analysis and the constrained analysis excluding *Welwitschia*, the Agaricomycetidae was resolved as significantly older than the Pinaceae, but in the constrained analysis retaining *Welwitschia *the 95% HPDs of node heights for Agaricomycetidae (+ Russulales) and Pinaceae are overlapping. Inspection of phylograms (Figure 6) reveals that *Welwitschia *has an elevated rate of molecular evolution compared with other gymnosperm lineages, suggesting that the deep conifer node in the constrained analysis including *Welwitschia *could be an artifact. On the other hand, the constrained analysis excluding *Welwitschia *implies that the gnepine clade (Pinaceae + gnetophytes) is younger than the angiosperms, which is surprising, considering that the oldest angiosperm fossils are Cretaceous in age, while the gnetophytes have a fossil record that extends to the Permian [32,33]. Given these conflicting lines of evidence, we consider the node heights for Pinaceae from the analyses including or excluding *Welwitschia *to be equally plausible.

**Table 1:** Median and 95% highest posterior densities (in parentheses) of node heights for selected clades of Agaricomycetes and seed plants inferred by Bayesian relaxed molecular clock analysis

Node	Unconstrained	Constrained, *Welwitschia *retained	Constrained, *Welwitschia *excluded
Agaricomycotina	0.199 (0.188–0.209)	0.195 (0.185–0.205)	0.195 (0.183–0.205)

Agaricomycetes	0.147 (0.138–0.155)	0.145 (0.137–0.154)	0.144 (0.135–0.153)

Agaricales/Polyporales	0.081 (0.076–0.085)	0.080 (0.075–0.084)	0.080 (0.074–0.084)

Agaricomycetidae	0.074 (0.070–0.078)	0.072 (0.068–0.075)	0.072 (0.067–0.076)

Agaricales	0.063 (0.059–0.067)	0.062 (0.058–0.065)	0.062 (0.057–0.066)

Boletales	0.052 (0.048–0.056)	0.052 (0.048–0.055)	0.051 (0.048–0.056)

Russulales	0.040 (0.034–0.046)	0.043 (0.037–0.049)	0.043 (0.037–0.049)

*Amanita*	0.033 (0.029–0.037)	0.033 (0.029–0.037)	0.033 (0.029–0.037)

*Laccaria*	0.040 (0.034–0.044)	0.043 (0.039–0.047)	0.043 (0.039–0.047)

Angiosperms	0.074 (0.069–0.079)	0.076 (0.071–0.080)	0.074 (0.069–0.079)

Rosids	0.046 (0.042–0.050)	0.045 (0.041–0.049)	0.044 (0.040–0.048)

Pinaceae	0.053 (0.048–0.058)	0.070 (0.065–0.074)	0.053 (0.048–0.058)

## Discussion

This is not the first molecular clock study to include both plants and fungi, but it is the first such study with sufficiently detailed taxon sampling to directly compare the relative ages of the nodes that are critical for reconstructing the evolution of ECM associations among the Agaricomycetes. The relaxed molecular clock method that we used is intended to compensate for heterogeneity in rates of molecular evolution, but this nonetheless remains a potential source of error, as illustrated by the impact of *Welwitschia *on node heights in gymnosperms. It remains to be determined (perhaps through simulation analyses) whether the degree of rate heterogeneity in our dataset is likely to cause artifacts in BEAST analyses. This approach requires an implicit ASR of the ECM habit in angiosperms and gymnosperms, which is discussed below.

Approximately 33 families of angiosperms have ECM-forming species [34]. Many of the most diverse and ecologically dominant groups of ECM-forming angiosperms are within the rosids, such as Fagales, Salicaceae, Dipterocarpaceae, and Myrtaceae, but there are also ECM taxa scattered across the monocots, basal eudicots, and asterids (Table 2) [34]. It is most parsimonious to infer that the ECM habit has arisen repeatedly across these groups of angiosperms, possibly involving repeated transitions from arbuscular mycorrhizal (AM) associations [34]. Molecular clock analyses of angiosperms by Wikström et al [35] suggest that the rosids is older than any of the clades of ECM-forming angiosperms outside of the rosids (Table 2). The symbiotic status of the ancestor of the rosids is not clear, however. There are many AM taxa in the rosids [34], suggesting either that there have been multiple origins of ECM in rosids or multiple reversals to AM associations. Wikström et al [35] estimated that the Fagales are about 60 to 61 million years old, while the entire rosid clade is about 100 to 109 million years old. Thus, the root node of the rosids provides a reasonable maximum age for the origin of the ECM habit in angiosperms, and it probably does not dramatically overestimate the age of origin of ECM in angiosperms.

**Table 2:** Divergence dates of angiosperm clades containing ectomycorrhizae-forming species (or species that form associations with ectomycorrhizae fungi, for example, Orchidaceae), based on Wang and Qiu [34] and Wikström et al [35]

Classification	ECM-containing group	Node	Age
Monocots	Orchidaceae	495	53–69

	Cyperaceae	434	28–39

	Poaceae	437	35–44

Basal Eudicots	Ranunculaceae	410	65–85

Caryophyllids	Polygonaceae	367	37–52

	Caryophyllaceae	382	28–40

	Nyctaginaceae	376	21–28

Asterids	Rubiaceae	304	61–64

	Oleaceae	260	55–64

	Aquifoliaceae	252	63–72

	Apiaceae	229	41–45

	Campanulaceae (+Lobeliaceae)	233 248^a^	82–90 46–59

	Goodeniaceae/Asteraceae	238 239^b^	65–69 44–50

	Caprifoliaceae	221 219^c^	29–36 54–58

Core Eudicots inc. sed.	Grossulariaceae	199	73–81

Rosids inc. sed.	Myrtaceae/Melastomataceae	121	78–88

Rosids 1	Euphorbiaceae	63	69–71

	Salicaceae	29	60–63

	Cunoniaceae	67	64–66

	Polygalaceae	85	66–68

	Fabaceae	83^d^	56–68

	Rosaceae	117^e^	46–47

	Rhamnaceae	105	62–64

	Ulmaceae	110	55–57

	Fagaceae + Juglandaceae + Betulaceae + Casuarinaceae	89^f^	60–61

Rosids 2	Dipterocarpaceae/Cistaceae	149	51–58

	Malvaceae	154	54–58

	Sapindaceae	139	20–26

'Node' refers to node numbering in Wikström et al [35]. Nodes are the most recent common ancestor of the ectomycorrhizae (ECM)-containing family and its closest non-ECM-forming relative, unless otherwise indicated. Two nodes are indicated when relevant taxa sampled by Wikström et al [35] are not in Wang and Qiu [34], or when topologies conflict. Ages in millions of years are ranges of ACCTRAN and DELTRAN optimizations from Wikström et al [35].

^a^MRCA (most recent common ancestor) of (Campanulaceae, Lobeliaceae)

^b^MRCA of (Asteraceae(Goodeniaceae, Calyceraceae))

^c^MRCA of (Valerianaceae(Linnaeaceae(Caprifoliaceae, Dispacaceae)))

^d^MRCA of Fabaceae

^e^MRCA of Rosaceae

^f^MRCA of Fagales

Within the gymnosperms, the overwhelming majority of ECM taxa are found in the Pinaceae, which are all (so far as is known) capable of entering into the symbiosis [34]. Several species of *Juniperus *(Cupressaceae) and one species of *Wollemia *(Araucariaceae) have also been reported to form ECM [36-38], but these reports are controversial [39]. Among the gnetophytes, only *Gnetum *has been shown to be ECM forming [34,40]. Based on published gymnosperm phylogenies [23,24], it is most parsimonious to infer that the ability to form ECM is a synapomorphy of the Pinaceae, whereas it is a derived condition within the Gnetales, Cupressaceae, and Araucariaceae (if it actually occurs in the latter taxa). Therefore, the maximum and minimum ages for the origin of ECM in gymnosperms are set by the split between the lineages leading to gnetophytes and the Pinaceae, which must have occurred by the Permian, about 270 Ma, and the diversification of Pinaceae, which began by the early Cretaceous, ca. 130 Ma [34,41].

To estimate the minimum number of origins of ECM symbioses in Agaricomycetes, we mapped the node heights of the rosids and Pinaceae onto the phylogeny of the Agaricomycetes [4,10,26]. All of our analyses indicate that the Agaricomycetes is too old to have been plesiomorphically mycorrhizal (Figures 1 and 3; Table 1). Our results also suggest that ECM symbioses involving rosids must have evolved independently in each of the eight major clades of Agaricomycetes that contain ECM-forming species (Figure 1). The minimum number of origins of ECM associations involving Pinaceae is more ambiguous. The constrained analysis that retained *Welwitschia *suggests there must have been at least six origins of ECM involving gymnosperms. Under this set of node heights, it is possible that the common ancestor of Agricomycetidae and Russulales could have been associated with a member of the Pinaceae lineage. The other two analyses, however, suggest that there must have been at least eight origins of ECM associations with gymnosperms, just as with angiosperms (Figure 1).

Our findings are consistent with prior studies that have inferred multiple origins of ECM, with no reversals [1,2,5] and they clearly reject the view that the common ancestor of all Agaricomycetes was mycorrhizal, with saprotrophy being a generally derived condition [11]. Evaluation of the multiple-gains-and-losses hypothesis [3] is not so straightforward, however, in large part because we are unable to reject the hypothesis that the ancestor of the Agaricomycetidae was mycorrhizal. Resolution of this problem would be aided by improved estimates of node heights in gymnosperms, which are obscured by evolutionary rate heterogeneity, particularly involving gnetophytes.

While our results suggest a minimum of six to eight independent origins of the ECM habit in Agaricomycetes, the actual number of transformations between ECM and saprotrophic lifestyles is surely much greater. Analyses of Matheny et al [5] and Binder and Hibbett [1] suggest that there were at least 14 independent origins of ECM in the Agaricomycetidae alone (11 gains in Agaricales and three gains in Boletales), and multiple lineages of ECM-forming and saprotrophic taxa are intermingled in the Russulales, Phallomycetidae, Cantharellales, and Sebacinales (it is most parsimonious to infer a single origin of ECM in the Thelephorales, and Hymenochaetales). Our taxon sampling does not span the root nodes of the Phallomycetidae, Cantharellales, and Sebacinales, however, and the design of our study enables us only to reject hypotheses of homology of the ECM habit. Thus, while the relative node heights of Agaricomycetes and potential ECM hosts suggest that there have been at least six to eight origins of ECM in Agaricomycetes, we expect that the actual number of gains is higher, and we are unable to address the possibility of reversals to saprotrophy within derived clades of Agaricomycetes. Detailed resolution of the pattern of transformations within individual clades of Agaricomycetes will require ASR analyses, which could be constrained according to the results of the present study. For example, it seems reasonable to adjust the prior probabilities of the ECM condition in the ancestor of the Agaricomycetes and backbone nodes leading up to the Agaricomycetidae to low values, while a 'flat' prior on symbiotic status would be appropriate for Boletales, Russulales, or any other group of fungi that diversified after the origin of potential host lineages.

Our findings and the fossil record both suggest that the first ECM associations probably involved the Pinaceae, but there is no evidence for a general trend in the evolution of ECM associations from gymnosperm to angiosperm hosts. Indeed, the Boletales and Russulales each appear to be young enough to have been primitively associated with either rosids or Pinaceae (Figures 2 and 3). The Agaricales, in contrast, appears to be significantly older than the rosids, but *Laccaria *and *Amanita*, two independently derived ECM-forming lineages within the Agaricales [42], are each resolved as younger than the rosids (Table 1; Figures 3 to 5). Moreover, recent phylogenetic studies involving ECM-forming taxa in *Inocybaceae *(Agaricales), *Leccinum *(Boletales), and Hysterangiales (Phallomycetidae), have resolved scenarios in which the plesiomorphic hosts are angiosperms, with later switches to Pinaceae [42-44]. Most of the larger genera of ECM Agaricomycetes that have been investigated in detail have been shown to associate with diverse gymnosperm and angiosperm hosts [45]. Much of this diversity may be due to recent host-switching events, as shown in *Inocybaceae, Leccinum*, and the Phallomycetidae. At the same time, our molecular clock analyses imply that some contemporary ECM associations, involving both gymnosperm and angiosperm hosts, may be the direct descendants of partnerships that were established early in the evolution of relatively young groups of Agaricomycetes.

## Conclusion

ECM symbioses have had a profound impact on the ecology of forest communities and the diversification of plants and fungi. Reconstructing the evolution of ECM has been difficult, however. Phylogeny-based ASR analyses, which have yielded conflicting results in fungi, are sensitive to tree topology, taxon sampling, and assumptions about evolutionary processes. Paleobiology provides another line of evidence on the problem, but many major groups of ECM-forming fungi have poor or nonexistent fossil records, and lack of precision in taxonomic placements reduces the value of the few available fossils for calibrating molecular clocks.

The approach taken here is independent of both ASR and the fungal fossil record. Our intent was not to reconstruct the origins of ECM in Agaricomycetes in detail, but rather to put 'brackets' on plausible histories of the character. Our results reject the view that the ancestor of the Agaricomycetes could have been an ECM-forming species. ECM associations involving angiosperms have evolved independently in eight major clades of Agaricomycetes, most likely multiple times within several of these clades (including Agaricales and Boletales), and associations with gymnosperms have evolved independently in six to eight clades of Agaricomycetes. The possibility that the ancestor of the Agaricomycetidae was an ECM-forming species cannot be firmly rejected based on our results (because one of our three analyses suggested that the stem node of the Pinaceae could be older than the Agaricomycetidae), although that seems unlikely based on the phylogenetic distribution of ECM and non-ECM clades in both Agaricales and Boletales [1,5].

Looking forward, there are several promising avenues for improving understanding of the evolution of ECM symbioses in Agaricomycetes. Tree-based ASR approaches can now be modified based on our results; constraints can be placed on nodes that are too old to have been involved in ECM associations, and reconstructions that allow both gains and losses of ECM can be performed within relatively young clades. Additional molecular clock analyses are also needed; resolving the relative ages of the Agaricomycetidae and the Pinaceae (both stem and crown nodes) should be a priority. This study used uncalibrated molecular clocks, which are appropriate to address the relative ages of clades. However, it will eventually be necessary to perform calibrated analyses to place the organismal phylogeny in the context of geological history. It seems unlikely that the fungal fossil record will ever provide a rich set of minimum ages for diverse nodes, such as that available for angiosperms [32], but well-supported cases of vicariance coupled with the geological record could provide a basis for calibrating fungal molecular clocks.

## Methods

Our dataset contained published RPB1 and RPB2 protein sequences (1936 amino acids, aligned) and nuclear large and small subunit rRNA gene sequences (3435 nucleotides) from 69 species, including 42 fungi, 17 plants, and 10 other eukaryotes (Additional file 1). We conducted alignments for the primary dataset using ClustalX, then made manual adjustments and excluded ambiguously aligned regions using MacClade 4.0. We constructed XML files for BEAST analysis using BEAUTi v.1.4.4 and conducted Bayesian molecular clock analyses with BEAST v.1.4.2 and 1.4.4, using the uncorrelated relaxed clock with lognormal rate distribution [21,22]. We employed the WAG+I+G model with four rate categories for proteins (this was the optimal model selected with ProtTest 1.3 [46]), and the GTR+I+G model with four rate categories for nucleotides, with normally distributed priors for the parameters in models of molecular evolution, a Yule process tree model, and default values for all other settings.

We performed an unconstrained analysis and two topologically constrained analyses using BEAST. In the unconstrained analysis, we used two user-defined starting trees, one with gnetophytes nested within conifers and the other with gnetophytes as the sister group to seed plants (both analyses converged to the same topology). In the constrained analyses, we used a user-defined starting tree and deleted all of the operators from the BEAST XML file that effect topological rearrangements. The BEAST XML file for the unconstrained analysis has been uploaded as Additional File 2. We ran two to five independent MCMC chains for 3.56 to 4.0 million generations total in each analysis. We assessed convergence by inspecting the log likelihood distributions of individual chains in Tracer and typically discarded the first 10% of the states sampled prior to combining results of individual chains in LogCombiner v. 1.4.4 and visualizing trees in TreeAnnotator v.1.4.4. To assess the relative ages of clades, we compared the 95% HPD distributions for node heights (expressed in average numbers of substitutions per site), considering nodes with non-overlapping 95% HPD ranges to be significantly different in age.

To visualize unconstrained branch lengths, we performed non-clock ML analyses, using the RAxML 7.0.4 servers at the Vital-IT Unit of the Swiss Institute of Bioinformatics  and the CIPRES Portal v1.14 at the San Diego Supercomputing Center [47,48]. We performed RAxML analyses with or without the topological constraint used in the BEAST analyses, retaining *Welwitischia *in both cases. As in the BEAST analyses, we employed the WAG model for proteins and the GTR model for nucleotides. RAxML analyses used one hundred rapid bootstrap (RBS) replicates, followed by ML optimization. Among-site rate heterogeneity was modeled with the CAT approximation during RBS and the initial ML optimization, switching to the discrete-gamma model with four rate categories during the final ML optimization [48]. The dataset used in RAxML analyses, and the optimal tree and model parameters from the topologically unconstrained analysis have been uploaded as Additional File 3.

## Authors' contributions

DSH and PBM designed the study. PBM assembled the datasets and DSH conducted the analyses and wrote the paper. Both authors have read and approved the final manuscript.

## Supplementary Material

Additional File 1 **Table S1**. GenBank accession numbers and genome project sources for sequence data. Click here for file

Additional File 2 **BEAST XML file**. This file was used to perform the topologically unconstrained relaxed molecular clock analysis. Datafiles for constrained analyses included a starting tree and omitted all operators effecting tpological rearrangements (but not adjustments of branch lengths and lineage-specific evolutionary rates). Click here for file

Additional File 3 **RAxML dataset and optimal unconstrained tree and model parameters**. The alignment was used to perform constrained and unconstrained non-clock maximum likelihood analyses in RAxML. Model parameters and the optimal tree from the unconstrained analysis, and the topology for the constrained analysis are also included. Click here for file
