# Supplementary material for: The relative ages of ectomycorrhizal mushrooms and their plant hosts estimated using Bayesian relaxed molecular clock analyses
Source: BMC Biol. 2009 Mar 10;7:13. doi: 10.1186/1741-7007-7-13 (PMC2660285; doi:10.1186/1741-7007-7-13)
Supplement: Additional File 1 — Table S1. GenBank accession numbers and genome project sources for sequence data. [file 1741-7007-7-13-S1.doc]

Table 1 - GenBank accession numbers and genome project sources for sequence data

| FUNGI |  | RPB1 | RPB2 | 18S rRNA | 25S rRNA |
| --- | --- | --- | --- | --- | --- |
| Agaricales | *Amanita brunnescens* | AY788847 | AY780936 | AY631902 | AY631902 |
|  | *Amanita phalloides* | AY485639 | AY485609 | AF026631 | AY380359 |
|  | *Armillaria mellea* | AY788849 | AY780938 | AY787217 | AY700194 |
|  | *Baeospora myosura* | DQ435801 | DQ470827 | DQ435796 | DQ457648 |
|  | *Bolbitius vitellinus* | DQ435802 | DQ385878  (5_11) | AY705955 | AY691807 |
|  | *Cantharocybe gruberi* | DQ435808 | DQ385879 | DQ234546  DQ234547 | DQ234540 |
|  | *Coprinopsis cinerea* | Broad Institute^1^ | Broad Institute^1^ | M92991 | AF041494 |
|  | *Coprinus comatus* | AY857983 | AY780934 | AY665772 | AY635772 |
|  | *Laccaria bicolor* | JGI^2^ | JGI^2^ | AY654886 | AY700200 |
|  | *Lycoperdon pyriforme* | AY860524  AY860523 | AY218495 | AF026619 | AF287873 |
|  | *Marasmius alliaceus* | AY860525  AY860526 | AY786060 | AY787214 | AY635776 |
| Boletales | *Boletellus projectellus* | AY788850 | AY787218 | AY662660 | AY684158 |
|  | *Calostoma cinnabarinum* | AY857979 | AY780939 | AY665773 | AY645054 |
|  | *Hygrophoropsis aurantiaca* | AY858961 | AY786059 | AY662663 | AY684156 |
|  | *Strobilomyces floccopus* | AY858964  AY858963 | AY786065 | AY662661 | AY684155 |
| Russulales | *Bondarzewia montana* | DQ256049 | AY218474 | AF026575 | DQ234539 |
|  | *Stereum hirsutum* | AY864885  AY864886 | AY218520 | AF026588 | AF393078 |
| Other Agaricomycetes | *Fomitiporia mediterranea* | AY864869  AY864870 | AY803748 | AY662664 | AY684157 |
|  | *Gautieria otthii* | AY864864  AY864865 | AY218486 | AF393043 | AF393058 |
|  | *Grifola sordulenta* | AY864877  AY864879 | AY786058 | AY665780 | AY645050 |
|  | *Hydnum repandum* | EF014376 | AAS67513 | AF026641 | AY700199 |
|  | *Phanaerochaete chrysosporium* | JGI^3^ | JGI^3^ | U59084 | AF287883 |
|  | *Tremellodendron pallidum* | DQ521411 | DQ408132 | AY766081 | AY745701 |
| Tremellomycetes | *Cryptococcus neoformans* | AAEY01000028 | AY485620 | X60183 | L14068 |
| Ustilaginomycotina | *Tilletaria anomala* | DQ234571 | AY803750 | AY803752 | AY745715 |
|  | *Ustilago maydis* | EAK84769 | EAK83484 | X62396 | AF453938 |
| Pucciniomycotina | *Phragmidium sp.* | EF014377 | AY485630 | EF014363 | AJ715522 |
|  | *Sporobolomyces roseus* | JGI^4^ | JGI^4^ | DQ832235 | DQ832234 |
| Ascomycota | *Cladonia caroliniana* | ABG81901 | AY584684 | AY584664 | AY584640 |
|  | *Magnaporthe grisea* | XP362207 | XP362269 | AB026819 | AB026819 |
|  | *Neurospora crassa* | XP329293 | AF107789 | X04971 | AF286411 |
|  | *Pichia stipitis* | JGI^5^ | JGI^5^ | AB053235 | U75728 |
|  | *Saccharomyces cerevisiae* | X96876 | M15693 | J01353 | J01355 |
|  | *Schizosaccharomyces pombe* | NP595673 | D13337 | X54866 | Z19578 |
|  | *Taphrina deformans* | EF014374 | AY485633 | U00971 | DQ470973 |
|  | *Trichoderma reesei* | JGI^6^ | JGI^6^ | AF548102 | AY544649 |
| Other Fungi | *Allomyces macrogynus* | ABM26998 | ABM27015 | EF014364 | AY552525 |
|  | *Endogone pisiformis* | DQ294601 | DQ302776 | DQ322628 | DQ273811 |
|  | *Glomus mosseae* | ABM26996 | EF014400 | AY635833 | DQ273793 |
|  | *Monoblepharis macrandra* | AAK00312 | ABM27021 | EF014369 | AY652933 |
|  | *Mucor hiemalis* | ABM26994 | ABM27009 | AF113428 | AJ876783 |
|  | *Paraglomus occultum* | DQ294602 | DQ826038 | DQ322629 | DQ273827 |
| OTHER OPISTHOKONTS |  |  |  |  |  |
| Animals | *Caenorhabditis elegans* | NP500523 | NP498047 | AY268117 | X03680 |
|  | *Ciona intestinalis* | JGI^7^ | JGI^7^ | AB013017 | AF212177 |
|  | *Drosophila melanogaster* | NP511124 | NP476706 | M21017 | M21017 |
|  | *Homo sapiens* | NP000928 | NP000929 | K03432 | M11167 |
|  | *Mus musculus* | P08775 | NM153798 | X00686 | X00525 |
| Mycetozoa | *Dictyostelium discoideum* | XP641735 | XP636812 | X00601 | X00601 |
| Choanoflagellates | *Monosiga brevicollis* | AAK00311 | ABM27024 | AF271999 | AY026374 |
| VIRIDIPLANTAE |  |  |  |  |  |
| Rosids | *Arabidopsis thaliana* | AL031986 | Z19121 | X52322 | X52322 |
|  | *Populus trichocarpa* | JGI^8^ | JGI^8^ | AY652861 | AF479118 |
| Other Angiosperms | *Amborella trichopoda* | AF519541 | AY699216 | U42497 | AY095449 |
|  | *Magnolia sp* | AF519539 | AF020841 | D29776 | AF389256 |
|  | *Nymphaea odorata* | AF519540 | AF043427 | AF206973 | AY095465 |
|  | *Oryza sativa* | XP493925 | XP_480298 | AF069218 | M11585 |
|  | *Rhododendron macrophyllum* | DQ020635 | AY566626 | AF419807 | AY727973 |
| Gymnosperms | *Cupressaceae sp*  *(Thuja/Cupressus)* | AY490550 | AY699211 | AF051797 | AY056503 |
|  | *Cycas revoluta* | AF519535 | AY563265 | D85297 | DQ008667 |
|  | *Ephedra sp* | AY490554 | AY699198 | U42493 | AY755719 |
|  | *Gingko biloba* | AY490553 | AF020843 | D16448 | AY095475 |
|  | *Pinaceae sp (Pinus/Pseudotsuga)* | AAQ08513 | AAW22921 | X75080 | AY056501 |
|  | *Taxus sp* | AY490551 | AAW22913 | EF017311 | AY056513 |
|  | *Welwitschia mirabilis* | AF519537 | AY699199 | AF207059 | DQ008662 |
|  | *Zamia sp.* | AF519534 | AAW22902 | M20017 | AY056481 |
| Other Viridiplantae | *Physcomitrella patens* | JGI^9^ | JGI^9^ | AF126289 | X80212 |
|  | *Chlamydomonas reinhardtii* | JGI^10^ | DQ020659 | AAY89365 | AF183463 |
| OTHER EUKARYOTES |  |  |  |  |  |
|  | *Cyanidioschyzon merolae* | BAC67672 | C. merolae Genome Project^11^ | AB158485 | AB158485 |
|  | *Phytophthora sojae* | JGI^12^ | JGI^12^ | AY742749 | X75631 |
|  | *Thalassiosira pseudonana* | JGI^13^ | JGI^13^ | AF374481 | AJ633506 |

^1^http://www.broad.mit.edu/annotation/genome/coprinus_cinereus.2/Home.html

^2^http://genome.jgi-psf.org/Lacbi1/Lacbi1.home.html

^3^http://genome.jgi-psf.org/Phchr1/Phchr1.home.html

^4^http://genome.jgi-psf.org/Sporo1/Sporo1.home.html

^5^http://genome.jgi-psf.org/Picst3/Picst3.home.html

^6^http://genome.jgi-psf.org/Trire2/Trire2.home.html

^7^http://genome.jgi-psf.org/Cioin2/Cioin2.home.html

^8^http://genome.jgi-psf.org/Poptr1_1/Poptr1_1.info.html

^9^http://genome.jgi-psf.org/Phypa1_1/Phypa1_1.home.html

^10^http://genome.jgi-psf.org/Chlre3/Chlre3.home.html

^11^http://merolae.biol.s.u-tokyo.ac.jp/

^12^http://genome.jgi-psf.org/Physo1_1/Physo1_1.home.html

^13^http://genome.jgi-psf.org/Thaps3/Thaps3.home.html
